# Sexual dimorphism in glutathione metabolism and glutathione-dependent responses

**DOI:** 10.1016/j.redox.2019.101410

**Published:** 2019-12-17

**Authors:** Luxi Wang, Yong Joo Ahn, Reto Asmis

**Affiliations:** Department of Internal Medicine, Wake Forest School of Medicine, USA

**Keywords:** Macrophage, Atherosclerosis, Obesity, Redox biology

## Abstract

Glutathione is the most abundant intracellular low molecular weight thiol in cells and tissues, and plays an essential role in numerous cellular processes, including antioxidant defenses, the regulation of protein function, protein localization and stability, DNA synthesis, gene expression, cell proliferation, and cell signaling. Sexual dimorphisms in glutathione biology, metabolism and glutathione-dependent signaling have been reported for a broad range of biological processes, spanning the human lifespan from early development to aging. Sex-depended differences with regard to glutathione and its biology have also been reported for a number of human pathologies and diseases such as neurodegeneration, cardiovascular diseases and metabolic disorders. Here we review the latest literature in this field and discuss the potential impact of these sexual dimorphisms in glutathione biology on human health and diseases.

## Abbreviations

AD: Alzheimer's disease
APAP: acetaminophen
Dio1: type I iodothyronine deiodinase
GCL: glutamate-cysteine ligase
GCS: γ-glutamyl cysteine synthase
GGCT: γ –glutamyl cyclotransferase
GGT: γ-glutamyl transpeptidase
GRX: glutaredoxin
GSH: glutathione
GSSG: glutathione disulfide
GPx: glutathione peroxidase
GR: glutathione reductase
GST: glutathione *S*-transferase
GSTA: glutathione *S*-transferase alpha
GSTM: glutathione *S*-transferase mu
GSTP: glutathione *S*-transferase pi
GSTP1: glutathione *S*-transferase pi 1
GSTT: glutathione *S*-transferase theta
MeHg: methyl mercury
MS: multiple sclerosis
PN: post-natal day
ROS: reactive oxygen species
SeGPx: seleno-dependent glutathione peroxidase

## 1 Introduction

**Fig. 1:**
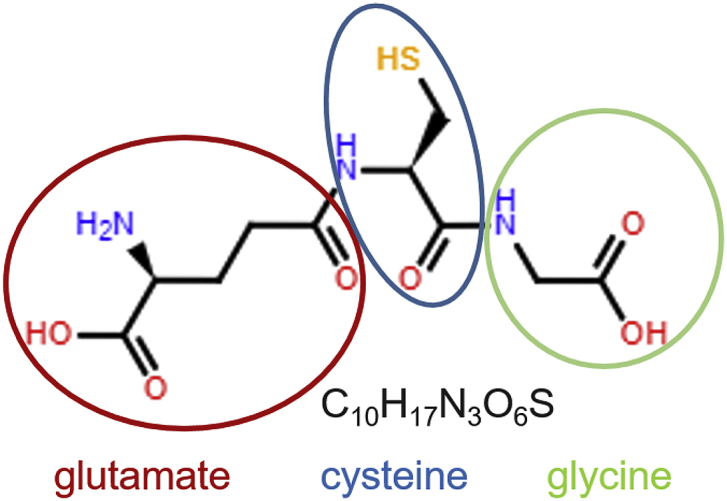
**Glutathione structure.** Glutathione (GSH) is a tripeptide consisting of three amino acids: glutamate (red), cysteine (blue) and glycine (green) and is present in most mammalian tissues and cells. GSH serves as a major antioxidant, reactive oxygen species (ROS) scavenger, detoxification agent and signaling molecule. (For interpretation of the references to colour in this figure legend, the reader is referred to the Web version of this article.)

Glutathione (GSH) is a tripeptide, γ-l-glutamyl-l-cysteinyl-glycine (Fig. 1), and the most abundant soluble thiol antioxidant and low molecular weight peptide in cells [86]. GSH is critical for the maintenance of redox homeostasis of cells and tissues, protects cells and tissues from oxidative and other forms of stress, and is intimately involved in the regulation of redox signaling pathways and detoxification reactions [31,55,86]. GSH functions include 1) detoxifying electrophiles; 2) eliminating reactive oxygen and nitrogen species; 3) maintaining the essential thiol redox status of proteins; 4) storing cysteine; 5) maintaining metal ion homeostasis and 6) modulating critical cellular processes, including DNA synthesis, microtubule-related processes, cell growth, proliferation and immune responses [18,49,69,75,126,129]. The ratio of reduced GSH to its oxidized form, glutathione disulfide (GSSG), serves a read-out of a cell's or tissue's thiol redox state [55,99], and even minor shifts in the GSH/GSSG ratio can have a dramatic impact on cellular functions, including proliferation, differentiation, cell growth and development, and survival [99]. Many biological processes and systems have shown sexual dimorphisms, including development and aging, mammalian phenotypic traits, social behavior, gut microbiome and the immune system (Elderman, de Vos, & Faas, 2018; Karp et al., 2017; Min et al., 2019; Pomatto, Tower, & Davies, 2017; Sato, Sando, & Takahashi, 1991; Xu et al., 2012). An increasing number of studies have reported that GSH metabolism and redox signaling also show significant sex differences. In this review, we will summarize the latest findings on sexual dimorphisms in GSH metabolism and GSH-dependent responses, and discuss the potential impact of these sex differences in neurodegeneration, cardiovascular diseases, cancer and metabolic disorders.

## 2 Glutathione synthesis, structure and function

**Fig. 2:**
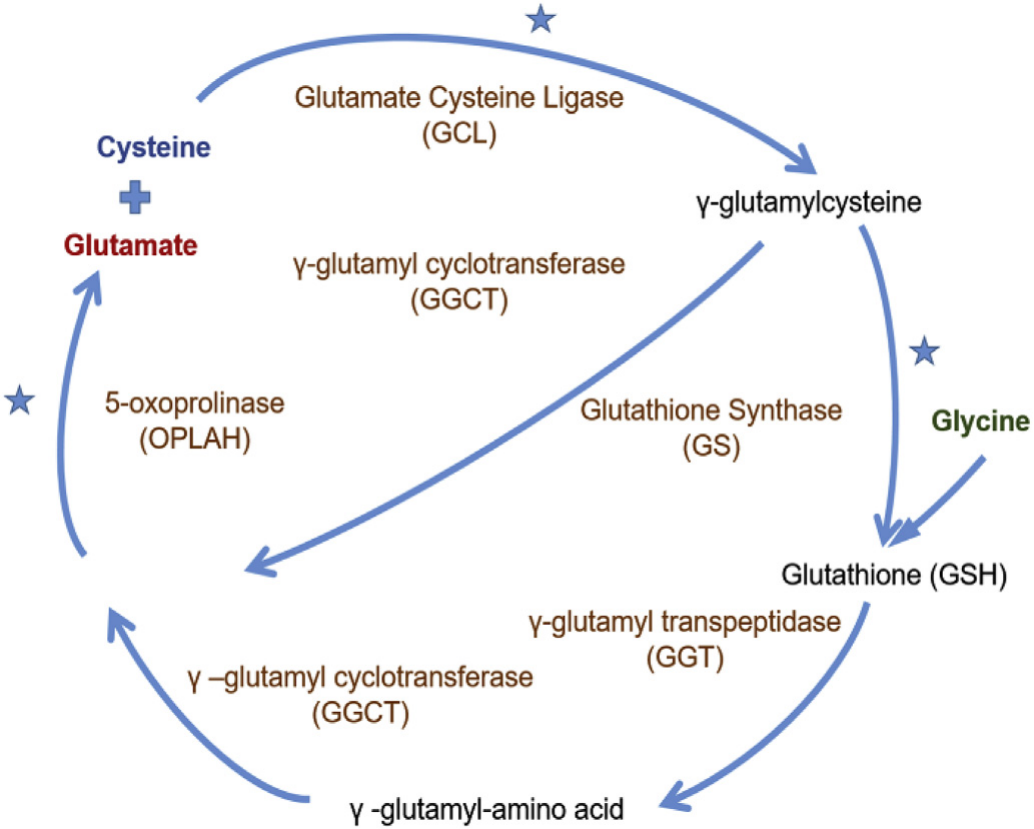
**Glutathione Synthesis and Metabolism.** The rate-determining step in GSH synthesis is the formation of γ-glutamyl cysteine is generated from cysteine and glutamate by the enzyme glutamate cysteine ligase (GCL). The addition of glycine to the dipeptide is catalyzed by GSH synthase (GS). GSH can only be degraded extracellularly by the membrane-bound enzyme γ-glutamyl transpeptidase (GGT), generating γ-glutamyl amino acids. γ-glutamyl-amino acids are transported via the blood stream, taken up by cells and tissues, and converted to 5-oxoproline in the reaction with γ –glutamyl cyclotransferase (GGCT). 5-Oxoproline is converted to glutamate and cysteine by 5-oxoprolinase (pyroglutamate hydrolase) with the energy input from ATP hydrolysis. * indicates an ATP-demanding step in the biosynthetic process.

**Fig. 3:**
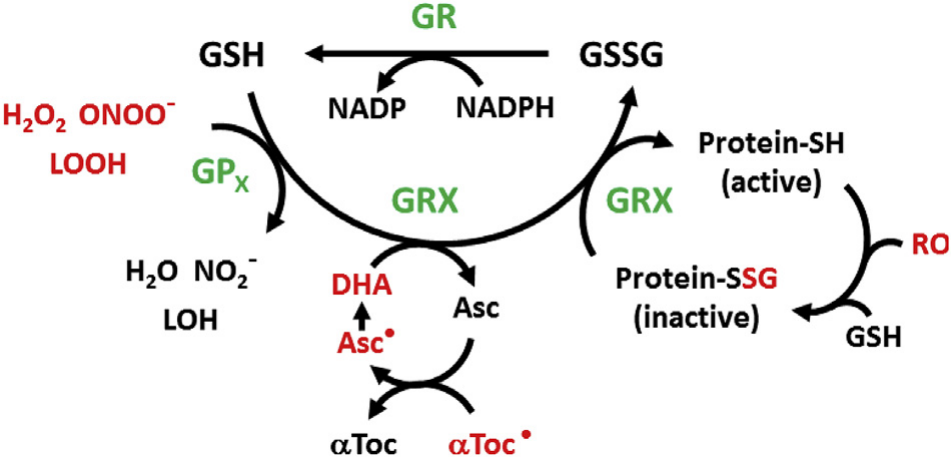
**Recycling of Glutathione by Glutathione Reductase.** Glutathione (GSH) is maintained in a reduced form by glutathione reductase (GR) in a NADPH-dependent manner. Enzymes are labelled in green. Glutathione peroxidases (GPx), glutaredoxin (Grx). (For interpretation of the references to colour in this figure legend, the reader is referred to the Web version of this article.)

Of the total cellular GSH, 80–85% is located in the cytosol, up to 15% in mitochondria and the remainder in the endoplasmic reticulum and the nucleus [70]. GSH concentrations range from 0.1 mM to 10 mM in the cytosol of cells, with around 1–2 mM in most cell types [85]. In hepatocytes, GSH concentrations can reach 10 mM as the liver is the major producer and exporter of GSH [31]. The structure of GSH was first established in 1935 by Harington and Mead [44]. Cellular GSH concentrations are controlled exclusively by GSH synthesis as cells cannot import GSH [38]. GSH synthesis requires glutamate, cysteine and glycine and occurs via two steps: the formation of the dipeptide γ-glutamyl-cysteine from glutamate and cysteine via glutamate-cysteine ligase (GCL, the ATP-requiring and rate-limiting step of GSH synthesis), and the subsequent addition of glycine via GSH synthase (Fig. 2) [31,38,70,71]. GCL transcription and activity are induced by oxidant stress and GSH depletion [70,117]. Mammalian GCL, formerly called γ-glutamyl cysteine synthase (GCS), is composed of two subunits: catalytic GCLC (73 kDa) and modifier GCLM (31 kDa) [37,46]. Within the cells, levels of reduced GSH are maintained by glutathione reductase (GR), which catalyzes the reduction of glutathione disulfide generated by the antioxidant enzymes glutathione peroxidases (GPx) and during the regeneration of oxidized glutaredoxins (GRX) (Fig. 3; see section 3.1). Due to the high demand in cysteine in the body, GSH acts as a continuous source and transporter of cysteine throughout the body [69,85].

Most cells do not degrade GSH, but they can export GSH into the extracellular milieu [70], where it is metabolized by γ-glutamyl trans-peptidase (GGT) on GGT-expressing cells [42]. The glutamate and cysteine of GSH are linked through the γ-carboxyl group of glutamate and can only be hydrolyzed by GGT (Fig. 2), which resides on the external membrane of cells [71,86]. The resulting dipeptide, cysteinyl glycine, can be taken up by cells via L-type amino acid transporters or further broken down to cysteine and glycine by dipeptidases in the plasma or extracellular fluids [45].

GSH plays key roles in the detoxication of xenobiotics, in antioxidant defense, and in cell signaling. It also regulates redox homeostasis in all tissues, including the brain [26]. Disruption of GSH metabolism such as impaired GSH synthesis or by GSH depletion, e.g. due to increased oxidative stress, is the major contributor to neurodegenerative diseases [3,102]. Noninvasive magnetic resonance spectroscopy showed that healthy young males have lower levels of GSH in the parietal cortical region of the brain than females [79], which may explain why men have a higher incidence of Parkinson's disease.

Sexual dimorphisms in GSH metabolism have also been reported in Wistar and Goto-Kakizaki rats (Table 1), a model of Type 2 diabetes [22]. The GSH/GSSG ratio in hepatic mitochondria of diabetic rats was lower in male than in female rats, indicating higher oxidative stress in male rats. Interestingly, in female rats the hepatic GSH/GSSG ratio can be increased by estrogen replacement therapy, suggesting that the protection of females against oxidative stress may be related by sex hormones. Moreover, the risk of Type 2 diabetes is also associated with genes encoding glutathione transferases (GST) in a sex-specific manner [7].

**Table 1:** Glutathione-related sex differences in humans and animal models.

Parameter	Tissue	Species/Animal Model	Condition	Difference	References
GSH concentration	erythrocytes	human	AD	F > M	[66]
GSH/GSSG ratio	Liver mitochondria	Wistar and Goto-Kakizaki rat	Type2 diabetes	F > M	[22]
GCL activity	liver	C57BL/6 mice	Liver damage	F > M	[84]
GGT activity	serum	human	Arterial tiffness	M > F	[54]

GR activity	liver	Fischer 344 rat	Aging	M > F	[92]
GR activity	brain	Sprague–Dawley rats	Developmental disorders	M > F	[28]
GR activity	liver	sheep	Fetuses	M > F	[1]
GR activity	endothelial cells	human	TBH challenge	F > M	[63]
GR activity	erythrocytes	human	Preterm infants	F > M	[41]
GPx activity	heart	rat	Castration	M > F	[11]
GPx activity	erythrocytes	human	Premenopausal period	F > M	[82]
GPx activity	heart	rat	Castration	M > F	[11]
GPx activity	erythrocytes	human	Preterm infants	F > M	[41]
GPx3 activity	kidney	mice	Orchidectomy	F > M	[91]
GST activity	brain	rat, vulture	Postnatal development	F > M	[20]
GST activity	brain	pigeon, kite, crow	Postnatal development	M > F	[20]
GST activity	brain	chicken	Healthy	M > F	[83]
GST activity	kidney	chicken	Healthy	F > M	[83]
seGPx activity	lung	human	Newborns	F > M	[116]

## 3 Sexual dimorphism of enzymes involved in GSH synthesis and metabolism

### 3.1 Glutamate cysteine ligase

During aging, GSH levels decline in bronchoalveolar lavage fluid in the lung and the cerebellum of male C57BL/6 mice but not in female mice [125]. Aging-related decreases in GSH content have been reported in many tissues and organs, including spleen, liver, and brain in both aged female and male rodents (Table 1) [68,88,135]. In the liver of male C57BL/6 mice, GCL protein levels are downregulated with age, and these changes correlated with the decline in GSH content of the spleen, lymphocytes and the brainstem [125]. Interestingly, these decreases in GSH content were more dramatic in male mice than female mice [67,68,125]. Female mice show significantly higher hepatic GCL activity than males [84]. Additionally, the GCL activity is more important in the protection of male C57BL/6 mice against acetaminophen (APAP)-induced liver injury than females, which have both higher basal activity levels and show stronger inducibility [14,84]. Furthermore, liver damage after APAP treatment due to the rapid depletion of GSH occurred only in livers of male C57/B6-129/Sv mice, but not in females [19]. The slower glucuronidation and greater accumulation of APAP in livers of female mice compared to males may be related to the higher rate of APAP-GSH conjugation observed in females [27]. In a different study, the Kavanagh group reported that subchronic exposure to methylmercury (MeHg) also significantly increased the activity of GSH synthesis and GCLC in the brains of female mice; unfortunately, no data from male animals were reported by the authors [115].

Sex differences in GSH metabolism have been reported in several neurodegenerative diseases, including Alzheimer's disease (AD) and Parkinson's disease. The GSH concentration in red blood cells was decreased in male AD patients compared with healthy controls but no differences have been found in females (Table 1) [66]. The decline in GSH concentrations in men was associated with a reduction in GCL and GS activity and impaired GSH synthase, but not lower GSSG levels or reduced GR activity, indicating that the loss of GSH in red blood cells of male AD patients is due to reduced GSH synthesis rather than impaired recycling of GSSG.

### 3.2 Glutathione reductase

GR catalyzes the reduction of GSSG to GSH, using NADPH as an electron donor (Fig. 3) [86]. GR is responsible for maintaining the GSH/GSSG ratio by reducing GSSG generated during states of oxidative stress. The ratio of GSH/GSSG is a sensitive indicator of changes in a cell's thiol redox state and ongoing redox signaling [53]. The GSH/GSSG ratio is remarkably similar across different cell types, but varies between different cellular compartments, and the changes of GSH/GSSG ratio affect cell functions such as apoptosis, differentiation and proliferation [53,57]. GSSG production increases during the detoxification of reactive oxygen species (ROS) by GPx and during the reactivation of oxidized GRX (Fig. 3; see section 3.1**)** [4], resulting in a decrease in the GSH/GSSG ratio [6]. Since GSSG increases can be harmful to cells, the reduction of GSSG by GR is essential for restoring the redox homeostasis [77].

Cytosolic GR activity is higher in liver supernatant fractions from male Fischer 344 rats than those obtained from females [92]. This difference is maintained during most of their adulthood but was not observed in aged rats. In male rats, concentrations of hepatic vitamin E, the most important antioxidant in cell membranes, are lower than in females but increase during aging. Whether this increase is due to increased GRX recycling, which controls vitamin E regeneration, is not known (Fig. 3; see section 3.1). Interestingly, female livers show age-dependent declines in GR activity. No such changes were observed in male liver cytosolic GR activity during aging [92]. A decline in GR activity was also observed in brain cortex homogenates during early post-natal development in both male and female rats [28], but no significant sex differences were observed. Sex-dependent differences in GR activity have also been reported in fetal sheep liver [1], male livers showing significantly higher GR activity.

### 3.3 Glutathione transferases

The superfamily of GST is composed of nearly 100 sequences and located on at least seven chromosomes [109,111]. GST play key roles in cellular detoxification against oxidative stress, xenobiotics and noxious compounds [109]. Based on the amino acid sequence similarity, tertiary structure, immunological function and kinetic properties, GST have been grouped into 13 classes, the best characterized being cytosolic GST, including alpha (GSTA), mu (GSTM), pi (GSTP) and theta (GSTT) [5,107]. Sexual dimorphisms with regard to GST have been observed in multiple organs, species and disease models.

Brain glutathione transferases are essential for the conjugation and binding of the neurotoxin acrylamide [24]. The activity of GST was higher in the brains of females than males, both in mice and in rats (Table 1) [20]. However, most birds showed the opposite trend for brain GSTs activity, being higher in male pigeons, kites and crows than females [20]. Vultures were the only birds with higher GST activity in females than males, which in the brain showed no detectable GST activity. The authors [20] suggest that the gender-specific GST activity differences may be related to the hormonal status of the animals, however, the study provided no evidence to support this hypothesis. The differences in GST activities between rats and birds have also been reported in a second study [83]. Higher renal GST activity in females compared to males has been reported in chickens, but interestingly the reverse was true for brains where male chickens actually showed higher GST activity than females (Table 1) [83]. The biological functions for these sexual dimorphisms in GST activity are unclear but they may be related to genetic variation between species.

Among all the GST, GST of the pi class are the most ubiquitous and the most prevalent GST isoforms in the brain [111,127]. The GSTP1 C341T polymorphism is found with a higher frequency of the C-T-transition in healthy subjects than patients with multiple sclerosis (MS); but females showed higher frequencies of the C-T-transition than males in both groups [9]. Also, male MS patients have lower GSTP1 activities and higher MS severity scores compared with females MS patients, suggesting a potentially critical detoxification role of GST in MS patients. These findings also suggest that GSTP1 detoxification activity is sex-dependent and may contribute to disease severity in male MS patients [9].

All major classes of GST isoenzymes are present at high levels in the kidney of mammals. In mouse kidneys, most GST isoforms are expressed at higher mRNA levels in females than males [30,58]. Among them, GSTA mRNA and protein expression were significantly higher in female mice kidneys (>2000-fold for mRNA) than in males [30]. GSTM2 and GSTO1 too showed higher expression in females than males [58]. The GST subunits 3 and 4 are highly expressed in rat kidney, and again females showed a 3-4-fold higher protein expression than males [16]. Conversely, the subunits 1 and 2 are expressed at higher levels in the kidneys of male rats. Sex differences in GSTM expression, a kidney injury biomarker, have been reported in Fischer 344 rats. Microarray analysis revealed that GSTM mRNA is expressed at 2-fold higher levels in kidneys of male rats than females [62].

In the gentamicin-induced urinary injury Sprague-Dawley rat model, already at baseline, male rat urine showed a higher protein level of GSTA than urine from female rats [35]. However, after 11 days of gentamicin treatment, female rats showed a 33-fold increase in GSTA activity compared to only a 21-fold increase in male rats. The more pronounced accumulation of GSTA in females correlates with histopathological findings showing more severe kidney damage in female rats. These findings suggest that GSTA may also be a potential biomarker renal injury.

### 3.4 Gamma-glutamyl transpeptidase

GGT is a cell surface enzyme that plays an important role in maintaining cysteine levels and glutathione homeostasis in the body [133]. The expression of GGT is upregulated in response to oxidative stress [132]. GGT is a well-established predictive biomarker for liver dysfunction and biliary tract diseases, and an indicator of pancreatic and bone diseases [59,73]. More recent studies have implicated higher GGT activity in arterial stiffness and cardiovascular diseases, and elevated GGT activity has been recognized as a predictive biomarker for atherosclerosis, arterial plaques heart and failure, but it is unclear whether this association is gender-dependent [54,59,81,89,97,110,134]. In a population-based cross-sectional study of 912 Japanese, serum GGT levels were associated with brachial-ankle pulse wave velocity in women, but not in men, and correlated with fatty liver disease and menopausal state [32]. A similar association between baPMV and serum GGT levels in women, but not men, had been reported in an earlier Korean study with 1387 participants (741 men, 646 women) [110]. Serum GGT activity and brachial-ankle pulse wave velocity have also been identified as cardiovascular risk factors in Chinese patients with coronary artery disease, but in this study for both genders [134]. A more recent Korean study reported that GGT activity is associated with an increased level of arterial stiffness in both genders [54,89]. This association, however, appears to be stronger in males than females (1.63; 95% CI, 1.21–2.20 versus 1.56; 95% CI, 1.08–2.27) [54]. In a four-year follow-up of the Korean Genome Epidemiology Study, Ha and colleagues showed that serum GGT levels correlate with blood pressure, but the correlation was only significant in men, not in women [40]. Serum GGT levels have also been shown to be associated with increased risk of mortality in patients with hepatic steatosis. An eleven-year longitudinal population study in Germany with 2044 men and 2116 women showed that in male, but not female patients with hepatic steatosis, GGT serum levels are a predictor of mortality [43].

The seemingly divergent findings with regard to GGT levels in women and men with cardiovascular diseases may be related to age and menopausal status of the populations in these studies. Ruttmann and colleges showed in an epidemiological study of 163,944 Austrian adults that younger participants (<60 years of age) show a stronger relationship between serum GGT and risk of CVD [96]. The hazard ratios for GGT levels and mortality in younger men and women were 2.03 and 2.6, respectively, but decreased to 1.42 and 1.52, respectively, in older men and women (>60 years of age) [96]. A similar finding was reported in a long-term epidemiologic study with 283,438 patients at the General Hospital of Vienna. Participants younger than 30 years of age with GGT concentration above the normal range showed higher hazard ratios than individuals older than 80 years of age [56].

A sexual dimorphism in GGT activity was also observed in mice and was linked to sex hormones [112]. The authors showed that in response to methylmercuric chloride treatment, renal GGT activity gradually increased in males but not in females. However, when females were treated with testosterone, GGT activity increased in response to methylmercuric chloride and now reach levels found in methylmercuric chloride-treated males, indicating that sex hormones contribute to the sex-differences in GGT activity found in mice.

## 4 Sexual dimorphism of GSH-Dependent antioxidant defenses and redox signaling

### 4.1 Glutathione peroxidases

GPx catalyzes the reduction of hydrogen peroxide to water as well as the reduction of other noxious peroxides, including lipid peroxides, into the corresponding alcohols [72]. GPx has also been reported to reduce highly reactive peroxynitrite [106]. In these reactions, GSH serves as the electron donor, resulting in the formation of GSSG [53,76] (Fig. 3). The class of mammalian GPx currently encompasses 8 enzymes, GPx1-GPx8 [15]. Of these, GPx1-4 and GPx6 are selenium-containing GPxs, and GPx5, 7 and 8 are non-selenium congeners [80,94].

During mouse lung development, in the perinatal period, females show higher GPx1 transcription levels than males, suggesting they may be better protected against oxidative stress [116]. Additionally, this sex difference is recapitulated by GPx enzymatic activity in the blood of these animals. The lower GPx1 mRNA expression in males than in females lungs during the first 5-day of birth and lower blood GPx activity in these animals may be related to the increase in oxygen tension during birth; however, to date there is no evidence that these sex-specific differences in GPx1 are associated with altered pulmonary functions [116].

Interestingly, the sex differences in GPx1 mRNA expression levels are not replicated in the kidney of 5-week mice [91]. Serum GPx3 activity was higher in female mice than in age-matched males (1.3-fold) despite the lack of a sex difference in renal GPx3 mRNA levels [91]. Additionally, the higher serum GPx3 activity in young female mice parallels higher type I iodothyronine deiodinase (Dio1) expression levels in the kidney (1.8-fold higher than in males), but not in the liver, where males have higher Dio1 expression [91]. However, sexual dimorphism of serum GPx activity and renal Dio1 expression vanished in aged mice [100]. In a study investigating sex differences in redoxl homeostasis in mouse brains, the authors reported that GPx1 protein levels were actually lower in old females than males, suggesting the enzymatic activity of this antioxidant is connected to the reproductive life cycle [2].

While the majority of studies on aging have included a single-gender, recent studies in Wistar rats compared genders and found that females, the longer-lived sex, had lower oxidative stress and less mitochondrial dysfunction than males [12,122,123].

The same sexual dimorphism and age-dependence in GPx activity were observed in rats. However, the sex differences in GPx activity in rat livers were not observed until after the animals had reached sexual maturity, at which point females showed 80% higher GPx activity than males (Table 1) [90]. Sex differences in GPx activity have also been reported in rat heart homogenates. However, the hearts of male rats had higher levels of GPx activity compared with age-matched females [11]. GPx activity in intraperitoneal resident macrophages did not differ between male and female adult rats [8].

In humans, GPx activity appears to be regulated by sex hormones such as progesterone and testosterone [8,17,82]. The activity of erythrocyte GPx was found to be higher in premenopausal than healthy postmenopausal women (*P* = 0.0014) (Table 1). Also, during the premenopausal period, female GPx activity is significantly higher (*P* = 0.025) than in age-matched men, but this difference was no longer observed between postmenopausal women and age-matched men [82]. Finally, GPx activity was reported to be significantly increased and greater in the placentas of obese women with a male fetus than a female fetus, however, the reason for this difference is not known [29].

### 4.2 Glutaredoxins and protein *S*-Glutathionylation

GRX are small molecular weight thiol transferases and members of the thioredoxin superfamily [104]. This enzyme exists in multiple isoforms and has been reported in most living organisms [21]. GRX catalyzes the reduction of GSH- protein mixed disulfides. The resulting GRX-GSH mixed disulfide is reduced by GSH, generating active GRX and GSSG, which in turn is reduced by GR and NADPH to GSH (Fig. 3) [34]. Little is known about sex differences in GRX activity or expression levels. The lumbosacral cord of female mice has higher GRX1 mRNA and protein expression levels and greater enzyme activity than male mice [23].

Interesting insights have emerged from GRX knockout mice. Once female mice reach 6–8 months of age, GRX1 deficiency promotes monocytes dysfunction and reprogramming, accelerates weight gain, and hyperglycemia, and at 18 months of age, these mice even develop small atherosclerotic plaques even though only very minor increases in plasam lipids where observed [25]. In contrast, GRX1-deficient male mice in the same C57BL/6J genetic background showed a much more subtle metabolic phenotype mice and no atherogenesis. However, male GRX1 KO mice on a C57BL/6NJ background develop obesity, hyperlipidemia, and hepatic steatosis by 8 months of age [103], suggesting a more complex mechanism by which sex differences affect the metabolic phenotype induced by GRX1 deficiency.

Protein *S*-glutathionylation is a well-recognized redox signaling mechanism operating in a wide range of cells and tissues [87,105,113]. The sexual dimorphic protein *S*-glutathionylation has been reported in macrophages isolated from dyslipidemic, atherosclerosis-prone LDL-receptor-deficient mice, which may account for the increased rate and severity of atherogenesis in females in this mouse strain [120]. The underlying mechanisms remain to be elucidated, but GRX1 was shown to protect monocytes and macrophages against nutrient stress-induced dysfunction, suggesting a role for the innate immune system in these sexually dimorphic responses to high-calorie diets [119,120].

GRX2 was first cloned from human in 2001, is located in both the nucleus and mitochondria, and has 34% sequence identity with GRX1 [74]. It is a major contributor to total glutaredoxin activity in most cells, contributing about 80% based on the 2-hydroxyethyl disulfide assay [124,131]. GRX2 has multiple isoforms, GRX2a residing in mitochondria [47]. The deletion of GRX2 in mice only increased the ROS production in male mice mitochondria but not in females [78]. In contrast, the loss of GRX2 in mice altered proton leak-dependent and phosphorylating respiration in female liver mitochondria [78]. It is noteworthy, that overexpression of GRX2a also has detrimental effects on mitochondrial function, disrupting mitochondrial respiration and ATP synthesis, as evidenced by mice with macrophage-specific overexpression of GRX2a [130]. These studies suggest the sex differences in mitochondrial functions may at least in part be due to sex differences in GRX isoforms and activity.

## 5 Sexual dimorphism in GSH metabolism in development

Sex differences in GSH metabolism during early development can profoundly impact a large number of physiological processes and disorders in adults. The dysregulation of GSH metabolism and impaired GSH synthesis in the brain is a common feature in neurodegenerative diseases [102]. Decreases in total brain GSH and lower GSH/GSSG ratio were found in neurodevelopmental disorder and psychiatric disorders [36,51,93]. According to the World Health Organization, infant mortality is higher among boys than girls [128] worldwide. Glutathione synthesis and recycling are important in early human development as evidenced by the higher enzymatic activity of intraerythrocytic GPx and GR in preterm baby girls as early as 24 h after birth [41]. Cord blood samples from females showed higher GST activity than males, which positively correlated with G6PD levels in 300 newborns from Jordan [48]. Also, the incidence of severe G6PD deficiency was higher in males compared with females. A similar finding also reported in *in vitro* study. When endothelial cells are challenged with an oxidative stimulus (tert-butyl hydroperoxide), female-derived tissues showed a two-fold increase of GR activity, whereas no changes were observed in male-derived tissues (Table 1) [63].

The GPx protein expression was higher in the male cerebral cortex than females at postnatal day (PN) 30 of Sprague–Dawley rats [28]. However, this sex-specific difference in GPx expression is reversed when the rats become sexually mature at PN60, with higher G6PDH activity in females than males [28]. G6PDH has been shown to regulate GSH regeneration [61], suggesting a higher rate of glutathione synthesis and regenerating in female rats during development.

Unlike other species, in fetal sheep liver GR activity (*P* = 0.004) and GSSG levels (*P* = 0.003) were significantly higher and total GSH trended higher (*P* = 0.07) in males than in their female twins [1]. The higher total GSH (*P* = 0.018) and GSSG (*P* = 0.011) content in fetal livers of males compared to females were also found in skeletal muscle, indicating increase oxidative stress during fetal development of female sheep.

To study antioxidant responses to environmental contaminants during early development, researchers exposed mice to MeHg, a neurotoxin from the gestational to PN21 to mimic prenatal and neonatal periods of human [95]. The GPx activity was increased more strongly in the female cerebrum, than in males. However, the GPx mRNA did not change after the MeHg exposure in either gender. These findings indicate that the observed sex-specific differences may be due to posttranscriptional mechanisms.

Sex-specific differences in GSH metabolism during early development. have also been reported for the environmental toxin hexavalent chromium. Exposure to hexavalent chromium during pregnancy through occupational contamination can cause higher pregnancy abortion, low birth rate and neonatal death [52,114]. A sexual dimorphism was found in the correlation between Cr accumulation and decreased expression levels of GPx1 have been found in the human placenta [10]. Higher Cr accumulation and lower GPx1 mRNA and protein expression were detected in the placenta associated with male fetuses compared with females, suggesting accelerated oxidative stress with a gender bias toward the male sex.

Sex-specific GPx expression was reported during mouse lung development. GPx1 mRNA expression in BALB/c mice lungs is higher in females than males during the neonatal saccular period [116]. But in the same study, no sexual dimorphism has been found for GPx2, 3 and 4 mRNA expression. Also, female lung seleno-dependent glutathione peroxidase activity was greater than in male lungs during the saccular phase but no significant differences were observed during the neonatal saccular period.

## 6 Sexual dimorphism in GSH metabolism in aging

Redox homeostasis as it relates to aging and lifespan has been studied for decades. The sex-dependence of maximum and mean lifespan differences in many species is related to the regulation and production of ROS [13,123]. Aged female C57BL6 mice show lower GPx1 protein expression and a 20% shorter lifespan than males [2,118].

Hepatic GPx activity also varies with age and sex. GPx activity in female rats continuously increases after birth until 18 months of age [90]. Male rats also show an increase in hepatic GPx activity as they age, but the increase was much slower than females. Forty-five days after birth, female rats already show a 20% higher GPx activity than males, and this difference increases to 80% in young adult rats (4 months old) [90]. Additionally, a sex difference was observed in the rate of aerobic oxidation of GSH in the liver. Female rats showed 50% higher liver aerobic oxidation of GSH than males at day 55 and this difference was maintained until the rats reached 4 months of age [90]. These differences correlate with the observed age-related changes in hepatic GPx activity, suggesting that GSSG formation may be due to the increased reduction of peroxides by GPx as the animals age.

Decreases in total GSH levels, decreasing GSH/GSSG ratios, and lower antioxidant enzyme activities (GPx, GR and superoxide dismutase) were observed during aging in humans [60,65,98,108]. A reduction in these antioxidant defenses promotes oxidative stress and may, therefore, contribute to the onset and progression of neurodegeneration in these patients. Age-dependent changes in GSH metabolism have been reported to differ between men and women with young women having higher antioxidant defenses than young men [50]. However, these differences decreased once women reach menopause [39,121].

In AD patients, women showed more dramatic losses of steroid sex hormones as compared to men with AD, and these losses parallelled AD-related cognitive decline and accumulation of amyloid-ß [64]. In addition, GPx activity in the brain of female postmenopausal AD patients was higher than in male patients, with significant differences detectable in parietal tissues and cerebellum [101]. However, this sex difference in GPx activity is AD-specific since the same study found no sex difference in GPx activity in healthy control brains. Since GR activity was not upregulated in female AD patients compared to men, the brains of female AD patients may be more susceptible to oxidative damage associated with AD [102]. In support of this hypothesis, a significant decline in GSH content was observed in brains of middle-aged women (±56 years of age) compared to young women (±26 years of age) [79].

Young women are particularly resistant to amyloid-ß toxicity, possibly due to their higher levels of mitochondrial GSH [12,33]. In rats, females showed higher hepatic mitochondrial GSH levels and higher mitochondrial GPx activity than males, but hepatic mitochondrial GSH levels dropped to those found in males after females were ovariectomized. This loss of mitochondrial GPx activity was prevented by estrogen replacement therapy [12]. Mitochondrial GSH levels in the brain were also significantly higher in young (3-month-old) female mice than age-matched male controls, but these differences disappeared once the mice reached 20 months of age [33], confirming maybe a link between sex hormones and mitochondrial GSH levels.

## 7 Conclusion

Sexual dimorphisms with regards to GSH metabolism and GSH-dependent responses have been reported in many animal studies as well as in humans. The factors contributing to these sex differences are just beginning to emerge, but appear to involve genetic background, levels of oxidative stress and sex hormones. These sex differences in GSH biology appear to contribute to a wide variety of pathologies and diseases. Clearly, more studies are needed to further explore the mechanism underlying these sex differences and their consequences on human health.

## Declaration of competing interestCOI

The authors have no conflicts to report.
